# Biology of Tendon Stem Cells and Tendon in Aging

**DOI:** 10.3389/fgene.2019.01338

**Published:** 2020-01-16

**Authors:** Pauline Po Yee Lui, Chi Ming Wong

**Affiliations:** ^1^Headquarter, Hospital Authority, Hong Kong, Hong Kong; ^2^Department of Health Technology and Informatics, The Hong Kong Polytechnic University, Hong Kong, Hong Kong

**Keywords:** tendon-derived stem cells, tendon stem cells, stem cell niche, tendon aging, stem cell aging

## Abstract

Both tendon injuries and tendinopathies, particularly rotator cuff tears, increase with tendon aging. Tendon stem cells play important roles in promoting tendon growth, maintenance, and repair. Aged tendons show a decline in regenerative potential coupled with a loss of stem cell function. Recent studies draw attention to aging primarily a disorder of stem cells. The micro-environment (“niche”) where stem cells resided *in vivo* provides signals that direct them to metabolize, self-renew, differentiate, or remain quiescent. These signals include receptors and secreted soluble factors for cell-cell communication, extracellular matrix, oxidative stress, and vascularity. Both intrinsic cellular deficits and aged niche, coupled with age-associated systemic changes of hormonal and metabolic signals can inhibit or alter the functions of tendon stem cells, resulting in reduced fitness of these primitive cells and hence more frequent injuries and poor outcomes of tendon repair. This review aims to summarize the biological changes of aged tendons. The biological changes of tendon stem cells in aging are reviewed after a systematic search of the PubMed. Relevant factors of stem cell aging including cell-intrinsic factors, changes of microenvironment, and age-associated systemic changes of hormonal and metabolic signals are examined, with findings related to tendon stem cells highlighted when literature is available. Future research directions on the aging mechanisms of tendon stem cells are discussed. Better understanding of the molecular mechanisms underlying the functional decline of aged tendon stem cells would provide insight for the rational design of rejuvenating therapies.

## Introduction - Biological Changes of Aged Tendons

Aging is known to reduce the regenerative capacity and increase the susceptibility of tissues and organs, including tendons, to injuries. Both tendon injuries and tendinopathies, particularly rotator cuff tears, increase with tendon aging (Teunis et al., 2014; Albers et al., 2016). In a systematic review including 30 studies with 6,112 shoulders, the prevalence of rotator cuff abnormalities increased with age, from 9.7% in patients aged 20 years and younger to 62% in patients aged 80 years and above (Teunis et al., 2014). Lower extremity tendinopathy was also reported to be more prevalent among older patients (Albers et al., 2016).

### Structural Changes

Aged tendons exhibited structural, compositional and biomechanical changes. Aged tendons appeared yellowish (mucoid degeneration) (Zhang et al., 2016). Collagen fibers were less organized in aged mouse patellar tendons (Dunkman et al., 2013). Ultrastructural analysis of distribution of fibril diameters indicated an increase of a subpopulation of large fibrils in aged mouse patellar tendons (Dunkman et al., 2013) and Achilles tendons (Gehwolf et al., 2016). There were inconsistent findings regarding the change of vascularity and blood flow in tendon during aging, depending on the species, tendon types, and definition of aging. While there was no age-associated change in vascularity in equine superficial digital flexor tendons (aged 2 to 23 years) (Gillis et al., 1997), aged rat patellar tendons (20-month-old) were reported to be highly vascular (Zhang et al., 2016). An early human study reported a reduction of vascularity in the supraspinatus tendons with age (aged 20–70 years) (Brewer, 1979). While there was no change in the rate of blood flow in the flexor tendons with age in a rabbit study (Landi et al., 1983), there was a significant decrease in blood flow in the intratendinous region of rotator cuff of elderly subjects compared with younger subjects (Funakoshi et al., 2010). Rudzki et al. (2008) reported a significant decrease in blood flow in intact supraspinatus tendon in asymptomatic subjects older than 40 years compared with younger subjects after exercise. Non-tendinous tissues such as fatty and cartilaginous tissues as well as calcification were reported in aged tendons in both animal models and human (Iagnocco et al., 2013; Zhang and Wang, 2015; Gehwolf et al., 2016; Wood and Brooks, 2016; Zaseck et al., 2016). These non-tendinous tissues compromise tendon structure and reduce tendon’s biomechanical properties.

### Compositional Changes

Tenocytes are the major cell types in tendons. The cell number decreased; tenocyte dedifferentiated and senescent; and their proliferation and metabolic activity reduced with aging (Ippolito et al., 1980; Josa and Kannus, 1997; Tsai et al., 2011; Dunkman et al., 2013; Yu et al., 2013).

The expression of extracellular matrix (ECM) genes and ECM remodeling gene were significantly reduced in aged Achilles tendons (Kostrominova and Brooks, 2013; Yu et al., 2013; Gehwolf et al., 2016; Wood and Brooks, 2016). The mRNA expression of collagen types I, III, and V, elastin and proteoglycan 4 (lubricin) in rat tendons decreased with age though immunostaining showed no apparent differences in protein levels of collagen type I and type V (Kostrominova and Brooks, 2013). The mRNA expression of collagen type I was reported to be similar in rat Achilles tenocytes in young (2 months) and aged (24 months) groups but the mRNA expression and enzymatic activities of MMP-2 and MMP-9 was significantly higher while the mRNA expression of tissue inhibitor of metalloproteinase-1 (TIMP-1) and TIMP-2 were significantly lower in the aged group compared to the young group *in vitro* (Yu et al., 2013). The expressions of collagen type I and type III genes were reduced in aged mouse Achilles tendons (Gehwolf et al., 2016). The collagen content of aged mouse Achilles tendon was similar to their young counterpart (Gehwolf et al., 2016) but the level decreased in canine patellar tendon with aging (Haut et al., 1992). In a small clinical study involving 7 old men and 10 young men, the collagen concentration in the patellar tendon biopsies was lower in the old men compared to that in the young men with a similar physical activity level, supporting the reduction in collagen during the aging process (Couppe et al., 2009).

Proteoglycans are important for regulating collagen fibril assembly, fiber size, and fiber sliding as well as cellular functions. The age-associated changes of proteoglycans were inconclusive. Thorpe et al. (2016) reported no change in the mRNA expression of collagens and proteoglycans as well as protein and mRNA levels of matrix remodeling enzymes in equine superficial digital flexor tendons with age (3.3 ± 0.6 years versus 19.0 ± 1.7 years). There was also no difference in the mRNA expression of biglycan, decorin, fibromodulin, and lumican in the patellar tendons of aged mice (Dunkman et al., 2013). While there was no change in the levels of major matrix components with age, there was a reduction in protein levels of several less abundant small leucine-rich proteoglycans (fibromodulin, mimecan, asporin) in aged equine superficial digital flexor tendons (Peffers et al., 2014). One study reported significant lower total glycosaminoglycan, chondroitin sulphate, and dermatan sulphate in healthy human supraspinatus tendons with age, although there was no change in the relative proportion of different glycosaminoglycan types (Riley et al., 1994). The concentration of nonenzymatic cross-links was higher in the patellar tendon biopsies of aged men compared to that in the young men in a small scale clinical study (Couppe et al., 2009). The advanced glycation end-products (AGEs) adduct level in tibialis anterior tendons was also higher in aged compared to adult mice (Wood and Brooks, 2016).

### Biomechanical Changes

The biomechanical properties of aged tendon were reported to be inferior in animal and human studies. The viscoelastic properties and mechanical strength of aged equine and mouse tendons were reported to be lower than those of young tendons (Dudhia et al., 2007; Dunkman et al., 2013; Zaseck et al., 2016). The mechanical properties (maximum stress and modulus) of aged rat Achilles tendon decreased with increasing age (Pardes et al., 2017). While aging did not alter tendon mechanical properties during homeostasis, it impaired tendon healing and hence biomechanical properties of flexor tendon in mice (Ackerman et al., 2017). Aged flexor tendons showed similar mechanical strength (maximum load to failure and ultimate tensile stress) but was significantly stiffer (higher Young’s modulus and stiffness) compared to young tendons (Gehwolf et al., 2016). Aged human Achilles tendons were also stiffer compared to young tendons as shown by sonoelastography (Turan et al., 2015). Aged human patellar tendons had significantly lower elastic modulus and shear wave velocity compared to young tendons as indicated by shear wave elastography (Hsiao et al., 2015). In a systematic review of age-related changes of biomechanical properties of healthy Achilles tendon, its stiffness, and elastic modulus decreased in older compared to younger adults (Delabastita et al., 2018). The responses of human Achilles and patellar tendons to transverse strain was reduced by 2.5% for every 10 years of life (Dudhia et al., 2007). **Table 1** summarizes the biological changes of aging tendons.

**Table 1:** Summary of biological changes of aging tendons.

• Yellowish color
• Enlargement of a subpopulation of collagen fibril size and change of fibril size distribution
• Disorganized collagen fibers
• Inconclusive findings on blood flow and vascularity
• Ectopic formation non-tendinous tissues including fat, cartilage and bone tissue
• Decreased cellularity
• Dedifferentiation and senescence of tenocytes
• Reduced proliferation and metabolic activity of tenocyte
• Inconclusive findings on change of collagen and proteoglycan content and composition
• Accumulation of advanced glycation end-products (AGEs)
• Inferior biomechanical properties particularly viscoelasticity

Consequently, the structure, composition, and biomechanical properties of aged tendons are compromised and aged tendons are prone to higher risk of tendon injuries, re-injuries, and tendinopathy as well as treatment failure (de Jonge et al., 2011; Teunis et al., 2014; Salini et al., 2015; Albers et al., 2016). The odds of a rotator cuff tear increased by 2.69-fold for every 10 years of age (Fehringer et al., 2008). After injury, aged tendons require very long time for rehabilitation. The current treatment modalities fail to completely restore tendon function. A summary of biological changes of aging tendons is showed in **Table 1**.

### Tendon Aging as a Problem of Aging of Tendon Stem Cells and Their Niche

Recent studies draw attention to aging being primarily a disorder of stem cells with a decrease in the number and functional fitness of tissue-specific adult stem cells (Sharpless and DePinho, 2007; Fukada et al., 2014). Tendon stem cells have been recently identified in tendons of various species (Bi et al., 2007; Rui et al., 2010). Stem/progenitor cells isolated from tendon mid-substances of various species, called tendon-derived stem cells (TDSCs), showed standard mesenchymal stem cell (MSC) characteristics, with expression of typical MSC surface antigens, self-renewal, colony-forming, and tri-lineage differentiation potential (Rui et al., 2010; Lui and Chan, 2011; Lui, 2015). In addition, they are able to form tendon- and enthesis- like tissues when implanted *in vivo*. TDSC expressed higher Oct4 level, showed higher clonogenicity and proliferative, and differentiation capacity compared to BMSC (Tan et al., 2012). TDSCs expressed embryonic stem cell markers and are different from tenocytes which are terminally differentiated cells (Zhang and Wang, 2010a). Recent studies showed that the functions of TDSCs declined or skewed with age *in vitro*. An in-depth understanding of the molecular mechanisms controlling tendon stem cell aging is essential to inform the development of stem cell-based therapeutics that can slow, or even forestall age-associated degenerative changes as well as enhance repair of aged tendons.

Niche is a specialized dynamic micro-environment which regulates stem cells’ functions and fate. Aging of cellular and acellular components of the niche can alter stem cell functions. Numerous studies showed that exposure of stem cells to a young systemic environment restored the regenerative capacity of stem cells in various tissues in aged mice (Conboy et al., 2005; Ruckh et al., 2012). Transplantation of aged spermatogonial stem cells to the testes of young male mice was sufficient to maintain stem cell functions (Sato et al., 2011). These findings underscore a major role of aging of the niche in the functional decline of stem cells and suggest opportunities of reversing tissue aging by targeting the niche.

### Scope of Review and Search Strategy

This review aims to summarize the biological changes of tendon stem cells in aging after a systematic search of PubMed. Relevant factors of stem cell aging including cell-intrinsic factors, changes of microenvironment, and systemic changes of hormonal and metabolic signals are reviewed, with findings on tendon stem cells highlighted when literature is available. Future research directions on the aging mechanisms of tendon stem cells are discussed.

In this review, “TDSCs” refer to stem/progenitor cells isolated from tendon mid-substances *in vitro* while “tendon stem cells” refer to stem/progenitor cells residing *in situ* in tendon mid-substances. TDSCs are often used as *in vitro* model to understand the physiology of tendon stem cells residing *in situ* in tendon mid-substances. The properties of tendon stem cells may change due to cell isolation and *in vitro* culture conditions. “Tenocytes” refer to terminally differentiated cells in tendons.

For the studies on aged TDSCs, the PubMed database was searched with the keywords ((tendon[Title/Abstract]) AND (ageing[Title/Abstract] OR aging[Title/Abstract] OR age[Title/Abstract])) AND (stem cells[Title/Abstract] OR progenitor) on 25 Jan 2019 and updated on 22 Sep 2019 with no restriction in language and year of publication. The studies were selected after reviewing the titles and abstracts. Original studies investigating the biological changes of stem cells in tendons, either *in vitro* or *in situ*, in aging were included. A total of 74 articles was identified. Of these, 16 studies are eligible. Another two studies were identified by hand search. Therefore, a total of 18 studies were included in this review and all of them are on the biological changes of TDSCs of aged tendons. There were no studies on aging of tendon stem cells *in situ*. **Table 2** summaries the studies on the biological changes of TDSCs in aging.

**Table 2:** Summary of biological changes of tendon-derived stem cells (TDSCs) in aging.

Species	Major findings	References
Mice	Young TDSCs were cobblestone-shaped while the shape of aged TDSCs were heterogeneous. Aged TDSCs proliferated slower and expressed lower levels of stem cell makers (Oct-4, nucleostemin, Sca1 and SSEA-1). They showed reduced tri-lineage differentiation potential compared to young TDSCs. Moderate mechanical stretching (4%) increased while 8% stretching decreased the expression of nucleostemin in aged TDSCs. Moderate mechanical stretching also increased the expression of *Nanog* and tendon-related markers (*Col1* and *Tnmd*). 8% stretching increased the expression non-tenocyte-related genes (*Lpl*, *Sox9* and *Runx2*) while 4% stretching had minimal effects on these genes. Moderate treadmill running of aged mice increased the proliferation of aged TDSCs in culture, decreased lipid deposition, proteoglycan accumulation and calcification, and increased the number of nucleostemin-expressing TDSCs in tendons.	Zhang and Wang, 2015
Mouse	The expressions of tendon transcription factor (*Scx*, *Mkx*), ECM (*Col1a1*, *Col3a1*, *Dcn*, *Bgn*, *Fmod*) and ECM-remodeling (Lox, *Thbs1*, *Sparc*) genes were significantly reduced in aged tendon tissue and aged TDSCs isolated from the Achilles tendons. There was no significant difference in the fascicle diameters between young and healthy-aged tail tendons. Aged Achilles tendon was thicker but there was no difference in the fascicle diameters, cell density and total collagen content compared to their young counterparts. Aged Achilles tendon displayed a bimodal distribution, with a more pronounced separation of large and small diameter fibrils and less interfibrillar area, showing no change in fibril number when compared to young tendons whose fibril size was normally distributed. The average fibril diameter was larger for aged Achilles tendon compared to young tendons. The fibers were less well-oriented in aged tendon. Aged flexor tendons showed similar mechanical strength (maximum load to failure and ultimate tensile stress) but was significantly stiffer (higher Young’s modulus and stiffness) compared to young tendons. There was an accumulation of lipid droplets in aged Achilles and tail tendons, with an increased expression of adipogenic markers *(Pparg* and *Cebpα*) and reduced expression of β-catenin (regulator of adipogenesis).	Gehwolf et al., 2016
Mouse	Aged TDSCs (18-month old) showed lower collagen contractility than cells obtained from younger animals (6-, 9-, and 12- months old).	Yin et al., 2019
Rat	Both aged and young TDSCs expressed nucleostemin, Oct-4 and SSEA-4. Aged TDSCs showed slower proliferation rate, frequency and cell cycle progression compared to young TDSCs. The expression of tendon related marker genes (*Scx*, *Tnmd*) was reduced while adipocyte differentiation and adipogenic markers increased in aged TDSCs. Cited2 was downregulated while CD44 was upregulated in aged TDSCs.	Zhou et al., 2010
Rat	Aged TDSCs showed lower cell proliferation, migration and tendon-related marker expression (*Tnc*, *Tnmd*, Scx) than young TDSCs. Medium of young TDSCs under hypoxic culture condition enhanced the migration, proliferation and mRNA expression of tendon-related markers (*Tnc*, *Tnmd* but not *Scx*) as well as reduced senescence of aged TDSCs.	Jiang et al., 2014
Rat	miR-135a, which directly binds to the 3’-untranslated region of ROCK1, was significantly downregulated in aged compared with young TDSCs. Overexpression of miR-135a in young TDSCs suppressed senescence, promoted proliferation, and induced migration and tenogenic differentiation, whereas suppression of miR-135a in aged TDSCs had the opposite effects. ROCK1 mediated the effects of miR-135a in TDSCs as shown by gain-of-function and loss-of-function studies.	Chen et al., 2015b
Rat	Aged TDSCs were large, flat and heterogeneous in morphologies while young TDSCs were uniformly elongated. Aged TDSCs showed higher stiffness compared to young TDSCs.	Wu et al., 2015
Rat	Moderate treadmill running accelerated tendon wound healing compared to the cage control. Moderate treadmill running also increased the number, proliferation, stem cell marker expression (Oct-4, Nanog) and tendon-related marker expression (collagen type I, collagen type III, tenomoduline) as well as downregulated the expression of non-tenocyte genes (*Pparg*, *Col2* and *Runx2*) of TDSCs.	Zhang et al., 2016
Rat	Young decellarized ECM (DECM) preserved stem cell properties of aged TDSCs. DECM from young TDSCs enhanced the proliferation, mRNA expression of tendon-related marker (*Scx*, *Tnmd*), protein expression stem cell marker protein expression (Oct-4, SSEA-1) of aged TDSCs. The senescence-associated β-galactosidase activity of aged TDSCs was also decreased by young DECM.	Jiang et al., 2018
Rat	FOXP1 mRNA and protein levels were markedly decreased in the aged TDSCs. Overexpression of FOXP1 attenuates TDSCs aging, as shown by reduced senescence-associated β-gal staining and expression of senescence marker, p16^INK4A^. FOXP1 overexpression also restores the age-associated reduction of self-renewal, migration and differentiation of TDSCs. Conversely, knockdown of FOXP1 promoted senescence in young TDSCs. In addition, FOXP1 overexpression rescued decreased levels of E2F1, pRb and cyclin D1 in aged TDSCs.	Xu and Liu, 2018
Human	Aged tenocyte-like cells showed lower proliferation rate, colony forming ability, collagen type I secretion and osteogenic differentiation potential compared to their young counterparts. However, there were no significant difference in the surface expression of MSC markers (CD29, CD44, CD73, CD90 and CD105) as well as mRNA expression of ECM (collagen type I, collagen type III, osteocalcin, decorin), TGF-β1, TGF-β2, TGF-β3 between aged and young tenocyte-like cells.	Klatte-Schulz et al., 2012
Human	Aged/degenerated TDSCs exhibited deficits in self-renewal and colony-forming ability. They showed significant decline in proliferative activity and poor response to FGF2 and TGF-β1. However, their multipotency was retained. Aged/degenerated TDSCs showed earlier cellular senescence with upregulation of p16^INK4a.^ Microarray and gene ontology analyses revealed that differential expression of genes regulating cell adhesion, migration and actin cytoskeleton. Aged/degenerated TDSCs showed slower migration, higher actin stress fiber content and slower turnover of actin filaments. Analyses of microarray candidates revealed dysregulated cell-matrix interactions with lower gene expression of collagen type I and collagen type I-binding integrins α1, α2 and α11 but higher gene expression of fibronectin and fibronectin-binding integrins αv, β3 and β5. The ROCK1 and ROCK 2 mRNA and protein levels as well as activities increased in aged/degenerated TDSCs. Inhibition of ROCK1 and ROCK2 activities in aged/degenerated TDSCs with ROCK inhibitor restored the cell area, F-actin content and dynamics as well as migration.	Kohler et al., 2013
Human	Aging affected the number of TDSCs isolated and colony-forming ability of TDSCs. However, tri-lineage differentiation potential of TDSCs was not affected by age.	Ruzzini et al., 2014
Human	Pin1 mRNA and protein expression levels were significantly decreased during prolonged *in vitro* culture of human TDSCs. Overexpression of Pin1 delayed the progression of cellular senescence, increased telomerase activity and decreased level of the senescence marker, p16^INK4a^. Conversely, Pin1 siRNA transfection promoted senescence in TDSCs.	Chen et al., 2015a
Human	The expression of ephrin receptors EphA4, EphB2 and B4 and ligands EFNB1 were down regulated in aged TDSCs. Activation of EphA4- or EphB2-dependent reverse signaling could restore the migratory ability and normalize the actin turnover of aged TDSCs. However, only EphA4-Fc stimulation improved aged TDSCs’ proliferation to levels comparable to young TDSCs.	Popov et al., 2016
Human	CITED2 mRNA and protein expression levels were significantly higher in young TDSCs than in old TDSCs. Old TDSCs displayed lower cell proliferation and higher senescence compared with young TDSCs. High level of CITED2 protein expression in young TDSCs correlated with the downregulation of SP1 and p21 and upregulation of MYC, suggesting the mechanism by which CITED2 upregulates TDSC proliferation. TGFβ2 downregulated the expression of *CITED2* gene and knockdown of CITED2 abolished the effect of TGFβ2 on TDSC proliferation and senescence.	Hu et al., 2017
Human	Tenogenic differentiation capacity of TDSCs decreased significantly with advancing age. Aged TDSCs showed higher β-galactosidase activity and p16 and concurrently lower collagen type I concentration and expression of tendon-related markers (*Scx*, *Tnmd*, *Bgn*, *Dcn*, *Col1*, and *Col3*). Overexpression of p16 significantly inhibited tendon-related marker expression in young TDSCs and the inhibitory effect was mediated by miRNA-217 and Egr1.	Han et al., 2017
Human	The cell stiffness and size of aged TDSCs were higher than young TDSCs. The aged TDSCs also showed a denser, well-structured actin cytoskeleton which correlated with the augmented cell stiffness. Treating aged TDSCs with ROCK-inhibitor, reversed these age-related changes, and has rejuvenating effect on cell morphology and stiffness.	Kiderlen et al., 2019

## Characteristics of Aged TDSCs

### Phenotypes of Aged TDSCs

Young TDSCs were reported as spindle or cobble-stone shape in previous studies (Rui et al., 2010; Zhang and Wang, 2015). However, TDSCs isolated from aged human, mouse and rat tendons were reported to be heterogeneous, much larger and show a round and flattened appearance (Wu et al., 2015; Zhang and Wang, 2015). The stiffness of aged rat TDSCs was higher than that of young TDSCs (Wu et al., 2015; Kiderlen et al., 2019). The increase in size and irregular shape of aged TDSCs might be associated with the dense cytoskeleton organization, which could lead to an increase in both stiffness and viscosity (Wu et al., 2015; Kiderlen et al., 2019). Indeed, the actin stress fiber content was higher and the turnover of actin filaments was slower in TDSCs isolated from aged/degenerated human Achilles tendon compared to TDSCs isolated from young/healthy tendons (Kohler et al., 2013). There was also an increase in focal adhesion kinase complex protein paxillin and profound rearrangement of actin cytoskeleton with an increase in actin in aged mouse Achilles TDSCs (Gehwolf et al., 2016).

### Frequency, Proliferative, and Colony-Forming Ability of Aged TDSCs

Fewer TDSCs were isolated from aged tendons compared to young tendons (Zhou et al., 2010; Ruzzini et al., 2014). Aged TDSCs showed delayed cell cycle progression (Zhou et al., 2010), lower proliferation (Zhou et al., 2010; Klatte-Schulz et al., 2012; Kohler et al., 2013; Jiang et al., 2014; Zhang and Wang, 2015; Hu et al., 2017), lower proliferative response to fibroblast growth factor-2 (FGF-2), and transforming growth factor β 1 (TGF-β1) (Kohler et al., 2013) as well as lower colony-forming ability (Klatte-Schulz et al., 2012; Kohler et al., 2013) compared to their young counterparts.

### Stem Cell Markers and ECM Expression and Migration Ability of Aged TDSCs

Aged TDSCs showed lower expression of stem cell markers (Oct-4, nucleostemin, Sca-1, and SSEA-1) (Zhang and Wang, 2015) and tenocyte-related markers (Zhou et al., 2010; Jiang et al., 2014) as well as lower collagen type I secretion (Klatte-Schulz et al., 2012; Kohler et al., 2013) and higher fibronectin protein expression (Kohler et al., 2013) compared to young TDSCs. However, Klatte-Schulz et al. (2012) reported no significant difference in the surface expression of MSC markers (CD29, CD44, CD73, CD90, and CD105) as well as mRNA expression of ECM proteins (collagen type I, collagen type III, osteocalcin, decorin), TGF-β1, TGF-β2, TGF-β3 between aged and young human tenocyte-like cells. Zhou et al. (2010) reported no difference in stem cell markers (nucleostemin, Oct-4, and SSEA-4) between young and aged rat TDSCs. The aged/degenerated TDSCs were deficient in cell adhesion, migration and actin dynamics (Kohler et al., 2013; Jiang et al., 2014). Aged mouse TDSCs showed lower collagen adhesion and contractility compared to their younger counterparts (Yin et al., 2019). This is consistent with change of transcriptome, especially in genes regulating cell adhesion, migration, and actin cytoskeleton, in aged human Achilles TDSCs (Kohler et al., 2013).

### Differentiation Potential of Aged TDSCs

While two studies reported similar tri-lineage differentiation potential of young and aged human TDSCs (Kohler et al., 2013; Ruzzini et al., 2014), one study reported higher adipogenic differentiation potential of aged rat TDSCs (Zhou et al., 2010) and another study showed lower osteogenic differentiation of aged human tenocyte-like cells (Klatte-Schulz et al., 2012). Zhang and Wang (2015) reported reduced tri-lineage differentiation potential of aged TDSCs compared to their young counterparts. Han et al. (2017) showed that the tenogenic differentiation capacity, collagen type I concentration, and expression of tendon-related markers (*Scx*, *Tnmd*, *Dcn*, *Bgn*, *Col1*, and *Col3*) of human Achilles TDSCs decreased with advancing age. Treatment of human TDSCs with tarzarotene, a retinoic acid receptor (RAR) agonist, induced nuclear translation of scleraxis and inhibited spontaneous cell differentiation *via* histone methylation but not histone acetylation in RAR signaling, suggesting that histone methylation plays roles in the differentiation of tendon stem cells (Webb et al., 2016). Regulation of methylation status of aged TDSCs may help to restore the tenogenic differentiation potential of TDSCs and this requires further research.

## Intrinsic Cellular Deficits

### Cellular Senescence

Cellular senescence is a state in which a cell no longer has the ability to proliferate (Collado et al., 2007; Hoare et al., 2010). Senescent cells are irreversibly arrested at the G1 phase of the cell cycle and do not respond to various external stimuli, but they remain metabolically active (Collado et al., 2007; Hoare et al., 2010). Cellular senescence can be triggered by a number of cellular stresses, including oxidative stress, telomere dysfunction, non-telomeric DNA damage, epigenetic derepression of the *INK4a/ARF* locus, and oncogenic activation (Collado et al., 2007). Senescent cells are characterized by shortened telomeres, increased activity of senescence-associated β-galactosidase (SA-β-gal), increased expression of p16^INK4a^, p53, p15^INK4B^, and p21^WAF1/Cip1^, and histological changes (Collado et al., 2007; Hoare et al., 2010). Cellular senescence is a potent tumor suppression mechanism. It is seen in aged or damaged tissues, and is associated with reduced regenerative capacity with age. Similar to other tissue-specific stem cells, aged TDSCs displayed cellular senescence (Kohler et al., 2013; Jiang et al., 2014; Han et al., 2017; Hu et al., 2017). The expression of p21^WAF1/Cip1^ (Hu et al., 2017) and p16^INK4a^ (Han et al., 2017) was higher in human aged/degenerated Achilles TDSCs. In another study, upregulation of *p16^INK4a^* gene and protein expression was observed, but the gene expression of *p14*, *p21*, and *p53* were unchanged in age/degenerated human Achilles TDSCs (Kohler et al., 2013). Further studies are required to confirm this finding. p16^INK4a^ is a critical effector of cellular senescence. Many studies showed that the increase in the expression of p16^INK4a^ with aging was linked to the impairment of the number and/or function of adult stem cells (Janzen et al., 2006; Molofsky et al., 2006; Sousa-Victor et al., 2014) while abolishing p16^INK4a^ function enhanced the regenerative potential of stem cells (Shibata et al., 2007; Sousa-Victor et al., 2014). Indeed, overexpression of p16^INK4a^ inhibited tenogenic differentiation of young TDSCs (Han et al., 2017). Analysis of the mechanism revealed that the effect was mediated by miR-217 and its target EGR1 (Han et al., 2017). Silencing *p16* in aged satellite cells restored their quiescence and regenerative potential (Sousa-Victor et al., 2014). miRNA are epigenetic regulators of multiple biological activities including stem cell differentiation. Microarray analysis suggested that dysregulation of the Rho-associated protein kinase (ROCK) pathway might be a key player in TDSC aging (Kohler et al., 2013). The ROCK1 and ROCK 2 mRNA and protein levels as well as activities increased in aged/degenerated TDSCs (Kohler et al., 2013). Inhibition of ROCK1 and ROCK2 activities in aged/degenerated human TDSCs with ROCK inhibitor restored the cell area, F-actin content, and dynamics as well as migration (Kohler et al., 2013). This is consistent with the findings of another study which treatment with ROCK inhibitor restored the morphology and stiffness of aged human TDSCs (Kiderlen et al., 2019). miR-135a which targets ROCK1, was significantly downregulated in aged compared to young TDSCs (Chen et al., 2015b). Inhibition of miR-135a was reported to increase senescence of TDSCs (Chen et al., 2015b). Overexpression of miR-135a in young TDSCs suppressed senescence, promoted proliferation, and induced migration and tenogenic differentiation *in vitro* (Chen et al., 2015b), suggesting that miR-135a is a key regulator of ROCK1-induced cellular senescence. The reduced expression of *Tnmd* in aged TDSCs (Zhou et al., 2010; Han et al., 2017) may affect cellular senescence of TDSCs as knockdown of *Tnmd* in mouse tail TDSC augmented cellular senescence, reduced the gene expression of cyclin D1, and increased the gene expression of *p5*3 but has no effects on the gene expression of *p16* and *p21* (Alberton et al., 2015). However, it is important to note that the complete knockout of *Tnmd* was artificially created in the animal model which might be different from the reduced expression of Tnmd in aged TDSCs. miR124 suppressed collagen formation during tenogenic differentiation of human TDSCs *via* suppressing EGR1 expression while anti-miR124 promoted it, suggesting that miR124 might be a promising therapeutic target for TDSC and tendon aging (Wang et al., 2016).

Pin1 (peptidyl-prolyl cis-trans isomerase), an enzyme which regulates conformational changes of phosphoproteins, is an important enzyme necessary for healthy aging and prevention of age-related diseases. Deficiency of Pin1 in mice caused an early-aging phenotype (Driver and Lu, 2010). Overexpression of Pin1 was reported to delay the progression of cellular senescence, increase telomerase activity and decrease expression of p16^INK4A^ while Pin1 knockdown promoted senescence of TDSCs *in vitro*, confirming that Pin1 is also an important regulator of tendon stem cell aging (Chen et al., 2015a). The Pin1-mediated anti-aging signaling mechanism might offer an attractive therapeutic target for protection against tendon aging.

Forkhead box P1 (FOXP1) plays a crucial role in tendon stem cell aging. FOXP1 directly repressed transcription of *p16INK4A* in BMSCs (Li et al., 2017). It controlled MSC fate and senescence during skeletal aging (Li et al., 2017). A recent study showed that the expression of FOXP1 mRNA and protein levels decreased markedly in aged TDSCs. Overexpression of FOXP1 attenuated TDSC aging with a decrease in SA-β-gal staining and p16I^NK4A^ expression (Xu and Liu, 2018). It also rescued decreased levels of E2F1, pRb, and cyclin D1 and restored self-renewal, migration and differentiation in aged TDSCs (Xu and Liu, 2018). Conversely, depletion of FOXP1 by siRNA promoted senescence of young TDSCs.

CITED2 is a transcription factor implicated in the control of cell proliferation and cellular senescence. Aged human Achilles TDSCs were shown to display lower mRNA and protein expression of CITED2 as well as lower proliferation and higher cellular senescence compared to young TDSCs (Zhou et al., 2010; Hu et al., 2017). Moreover, TGF-β2 was reported to downregulate the expression of *CITED2* gene and knockdown of *CITED2* abolished the effect of TGF-β2–mediated TDSC senescence (Hu et al., 2017). The effects of TGF-β2 inhibitors and CITED2 activators in reversing the senescence of aged TDSCs and tendon aging await further studies.

### Accumulation of Damaged DNA and Proteins

Aging is one of the major risk factors for DNA damage, protein mis-folding and accumulation. Age-dependent deficits of the autophagy-lysosomal and ubiquitin-proteasome systems, which remove the damaged proteins, were reported in recent studies (Rubinsztein et al., 2011; Tomaru et al., 2012). However, other studies argued that it was the increase in damage caused by metabolic stress, not the decrease in autophagy potential and proteasome activity, which overwhelmed the protective capacity of proteolytic systems in aged cells (Warr et al., 2013). Inhibition of autophagy has been shown to inhibit self-renewal and differentiation of stem cells, including TDSCs (Cheng et al., 2015; Chen et al., 2016; Sbrana et al., 2016). Tendon aging is dependent at least in part on the actions of mechanistic target of rapamycin (mTOR). Long-term dietary administration of rapamycin, an inhibitor of mTOR, was reported to attenuate aged-associated increase in stiffness; decrease in elastiscity, cellularity, and progression of spontaneous tendon calcification; as well as enhance collagen turnover in aged mouse tendons (Wilkinson et al., 2012; Zaseck et al., 2016). Autophagy plays an important role in regulating the self-renewal capacity and stemness of human TDSCs through suppressing reactive oxygen species (ROS) production. Treatment of human TDSCs isolated from supraspinatus tendon of adult donors (26–34 years old) with starvation or rapamycin prevented H2O2-induced loss of self-renewal capacity and stemness (Chen et al., 2016). The effects were mediated by increasing autophagy activity and suppressing ROS production in TDSCs (Chen et al., 2016). On the other hand, inhibition of autophagy reduced the protective effects of starvation and rapamycin on H2O2-treated cells (Chen et al., 2016).

## Aged Niche

### Cell-Cell Communication

Stem cells interact with each other or niche cells *via* direct interaction of membrane proteins or secretion of soluble factors. The changes of membrane proteins or secretome of stem cells or niche cells during aging therefore could impact stem cell fate and functions. Ephrin are receptor tyrosine kinases for mediating short-distance cell-cell communication. Specifically, ephrins were reported to regulate the attachment, spreading, migration, differentiation, and age-associated senescence of stem cells (Arthur et al., 2011; Goichberg et al., 2013; Li and Johnson, 2013). Dysregulated cell-cell communication and hence proliferation, motility, and actin turnover of aged TDSCs were reported to be mediated by down-regulation of ephrin receptors EphA4, EphB2, and EphB4 and ligands EFNB1 (Popov et al., 2016). Interestingly, activation of EphA4- or EphB2- dependent reverse signaling in aged TDSCs restored migratory ability and normalized actin turnover while activation of EphA4 reverse signaling increased proliferation of aged TDSCs (Popov et al., 2016).

### Extracellular Matrix

The ECM of tendon consists primarily of collagens (mainly collagen type I), proteoglycans, and glycoproteins. ECM functions to provide mechanical strength to tendon. In addition, it also functions to retain resident cells in place, localize and create gradients of instructive signals that guide stem cells in their processes of self-renewal and differentiation.

The alterations in the composition, topography and organization of ECM of aged tendons therefore can contribute to the loss of functions of tendon stem cells. While tendon-derived decellularized matrix promoted tendinous phenotype and inhibited osteogenesis of TDSCs in the presence of osteogenic induction conditions, bone-derived decellularized matrix induced osteogenic differentiation of TDSCs (Yin et al., 2013). The accumulation of AGEs with age likely plays a significant role in stem cell dysfunction during tendon aging. AGEs are proteins or lipids that become nonenzymatically glycated and oxidized after exposure to carbohydrates. Proteins are usually glycated through the lysine resides. Collagen, which is rich in lysine, is frequently glycated. Non-enzymatic glycation of collagen type I diminished collagen-proteoglycan binding and weakened cell adhesion and migration (Reigle et al., 2008). The formation of AGEs also resulted in the denaturation and cross-linking of collagen and the AGEs were nearly irreversible once formed. The mechanical and biological functions of long-lived proteins like collagen are therefore affected in the aging process. AGEs also increased the activity of transglutaminase, a cross-linking enzyme of ECM proteins, in tenocytes *in vitro*, further promoting collagen cross-linking (Rosenthal et al., 2009). The collagen cross-links limited fiber sliding, reduced viscoelasticity of tendon, and hence increased its stiffness and brittleness (Reddy, 2004; Gautieri et al., 2017), consistent with the increase in tendon stiffness with age (Andreassen et al., 1981; Haut et al., 1992). Matrix stiffness is known to regulate proliferation and lineage commitment of MSCs (Engler et al., 2006; Nam et al., 2011; Lee et al., 2014; Navaro et al., 2015). Matrix with lower stiffness promoted chondrogenic differentiation, whereas matrix with high stiffness promoted osteogenic differentiation of stem cells (Nam et al., 2011; Navaro et al., 2015). The increase in matrix stiffness as a result of age-induced collagen cross-links might underlie higher non-tenogenic differentiation potential of aged TDSCs. Bone morphogenetic protein-2 (BMP-2) stimulated non-tenogenic differentiation and promote proteoglycan deposition of TDSCs *in vitro* (Rui et al., 2013). A recent study showed that AGEs increased BMP-2 expression and accelerated progression of atherosclerotic calcification in diabetes (Wang et al., 2012). AGEs were also reported to increase the mRNA expression of BMP2, BMP4, and osteogenic markers of human yellow ligament cells isolated from cervical spine (Yokosuka et al., 2007). There has been no study about the effects of AGEs on TDSCs and is an area worthy of investigation.

The ECM composition can affect tendon stem cell fate. A deficiency in proteoglycans (biglycan and fibromodulin) resulted in altered differentiation of TDSCs towards the osteogenic linage *via* modulating the BMP signaling (Bi et al., 2007). Secreted protein acidic and rich in cysteine (Sparc), also known as osteonectin, is a collagen-binding matricellular glycoprotein involved in collagen fibril assembly and procollagen processing. It regulates cell-ECM interactions impacting upon cell signaling, adhesion, proliferation, migration, and survival. Sparc also regulates adipogenesis by modulating cell adhesiveness and cytoarchitecture. A recent study reported that the expression of Sparc decreased in aged mouse Achilles tendons (Gehwolf et al., 2016). The decrease in Sparc expression led to a more contracted phenotype, an altered actin cytoskeleton, and an elevated expression of the adipogenic marker genes *Pparg* and *Cebpa* with a concomitant increase in lipid deposits in both aged and Sparc -/- mouse tendons, a phenomenon that is commonly seen in elderly (Gehwolf et al., 2016).

The cytoskeleton of a cell and cell-matrix interactions affect stem cell migration, proliferation, and differentiation. Cell migration is strongly dependent on fibronectin, actin cytoskeletal organization, and turnover rate of actin filament. Integrins bind stem cells to different ECM components and regulate downstream signaling which are important for stem cell self-renewal and proliferation (Piwko-Czuchra et al., 2009; Ellis and Tanentzapf, 2010). Aged/degenerated TDSCs showed lower actin turnover, gene expression of collagen type I, and collagen type I-binding integrins α1, α2, and α11 but higher gene expression of fibronectin, fibronectin-binding integrins αv, β3, and β5 compared to young/healthy TDSCs (Kohler et al., 2013). The changes therefore might cause poor cell adhesion, migration, and actin dynamics of aged/degenerated TDSCs (Kohler et al., 2013). Integrin receptors and their ECM ligands function upstream of RhoA/ROCK signaling. Both the expression and activities of ROCK1 and ROCK2, which mediate RhoA-induced actin stress fiber formation *via* phosphorylation of myosin light chain (Schmitz et al., 2000), increased in aged/degenerated TDSCs (Kohler et al., 2013). Inhibition of ROCK1 and ROCK2 activities in aged/degenerated TDSCs with ROCK inhibitor Y-27632 restored the cell area, F-actin content and dynamics as well as migration ability, with results comparable to young/healthy TDSCs (Kohler et al., 2013).

CD44 is a cell-surface glycoprotein involved in cell adhesion and migration. As the expression of CD44 is implicated in poor tendon healing (Favata et al., 2006; Ansorge et al.,2009), the increased expression of CD44 in aged rat TDSCs might contribute to reduced tendon repair capacity with age (Zhou et al., 2010). Overexpression and knockdown experiments of CD44 in aged TDSCs would shed light on the aging mechanisms.

Collagen fibers are poorly organized in aged tendons (Gehwolf et al., 2016). This is partly due to chronic-inflammation and alteration in the production and activities of MMPs in aged tendons (Yu et al., 2013). The change of organization of collagen fibers from an intact, tightly-packed parallel orientation to a more random arrangement in aged tendons could affect the fate of resident tendon stem cells. Human fetal TDSCs seeded onto randomly-oriented fibrous scaffold underwent osteogenesis with lower levels of integrin α1, α5, and β1 subunits compared to cells seeded onto aligned fibers (Yin et al., 2010).

### Locally Produced Biological Signals

Aging causes changes in the local production of biological signals at the stem cell niche which can profoundly impact tissue stem cell functions. Growth factors such as FGF, insulin growth factor-1 (IGF-1), vascular endothelial growth factor (VEGF), TGF-β1, platelet-derived growth factor (PDGF) and BMPs, as well as proteins in the Wnt/β-catenin, notch, and angiopoietin-1 signaling pathways are important modulators of stem cell functions. The Wnt/β-catenin and BMP signaling pathways enhanced non-tenogenic differentiation of TDSCs *in vitro* (Lui et al., 2013; Rui et al., 2013). Dysregulation of Wnt/β-catenin signaling pathway was observed in different aged stem cell and animal models (Brack et al., 2007; Liu et al., 2007; Gehwolf et al., 2016). However, there was no differences in mRNA expression of connective tissue growth factor (CTGF), TGF-β1, or stromal cell-derived factor 1 in tendons of old and young rats (Kostrominova and Brooks, 2013). The mRNA and protein expression of TGF-β1 also did not change with age in rat Achilles tenocytes (Yu et al., 2013). There was disruption of circadian control in aged mouse tendon (Yeung et al., 2014). One of the circadian genes, Gremlin-2 (Grem2) oscillated antiphase to BMP signaling and inhibited BMP-2 induced osteogenic differentiation of human tenocytes (Yeung et al., 2014). The study reported reduced Grem2 expression, dysregulated BMP signaling, and spontaneous calcification in aged mouse tendon, suggesting that inhibition of Grem2 might be involved in the calcification of aged tendons (Yeung et al., 2014). The growth factor profile of aged tendons and their effects on functions of tendon stem cells should be examined. Modulation of these growth factor signaling pathways might shed light on strategies to prevent tendon aging and promote healing of aged tendons after injury.

### Inflamm-Aging

Aging is associated with reduced ability of the body to modulate inflammation, resulting in chronic low-grade inflammation termed “inflamm-aging” (Franceschi et al., 2000). Aged mice were reported to show higher serum levels of inflammatory cytokines such as interleukin-6 (IL-6) and tumor necrosis factor α (TNF-α) compared to young mice (Wahl et al., 2010). There were greater inflammatory changes with higher IL-1βand IL-6 levels in flexor digitorum tendons of aged rats performing high repetition low force handle-pulling task (HRLF) compared to tendons in young rats performing HRLF and aged rats not performing HRLF (Kietrys et al., 2012). More macrophages and CTGF-immunoreactive fibroblasts were observed in the peritendon of supraspinatus tendons in aged rats after HRLF (Kietrys et al., 2012). The greater chronic inflammation in aged rat tendons after upper extremity overuse was associated with a decrease in forelimb agility and lack of improvement of success in reaching task (Kietrys et al., 2012). Dakin et al. (2012) measured the level of prostaglandin E2 (PGE2) in injured equine flexor tendons and found that the level of PGE2 increased while the level of formyl peptide receptor 2 (FRP2)/ALX, a receptor responsible for suppressing inflammatory response, decreased during injury with increasing age of the horses. Uninjured tendon explants isolated from younger (<10 years) but not older horses (≥10 years) treated with IL-1β responded by increasing FRP2/ALX receptor (Dakin et al., 2012), suggesting that aged tendons have lower capacity to resolve inflammation. de Gonzalo-Calvo et al. (2010) measured the oxidative stress and inflammatory cytokines of three volunteer groups, consisting of normoxic middle-aged people, normoxic aged people older than 75 years, and hypoxic aged people older than 75 years. The results showed that the antioxidant defenses of older people with hypoxia was lower. Serum TNF-α was significantly increased in the aged population while IL-6 was highly elevated in the hypoxic elderly population (de Gonzalo-Calvo et al., 2010). Another recent clinical study on 831 subjects showed that circulating mitochondrial DNA (mtDNA) increased with age and significantly contributed to the maintenance of chronic, low-grade inflammation observed in the elderly subjects with an increase in serum TNF- α, IL-6, RANTES, and IL-1ra (Pinti et al., 2014). Moreover, mtDNA values of siblings were directly correlated, suggesting a role for familiar/genetic background in controlling the levels of circulating mtDNA (Pinti et al., 2014). *In vitro* stimulation of monocytes with mtDNA resulted in an increased production of TNF-α, supporting that mtDNA could modulate the production of proinflammatory cytokines (Pinti et al., 2014). These findings supported that aged individuals exhibited reduced capacity to resolve inflammation and aging might scramble tendon stem cell functions and promote the development of chronic tendinopathy through these pathways. Indeed, high concentration of PGE2 was reported to reduce the proliferation and enhance non-tenocyte differentiation of TDSCs (Zhang and Wang, 2014). IL-1β irreversibly inhibited tenogenic and adipogenic differentiation as well as reversibly inhibited chondrogenic and osteogenic differentiation of TDSCs isolated from injured mouse Achilles tendons (Zhang et al., 2015). IL-6 was reported to stimulate rat Achilles TDSCs’ proliferation and entry into cell cycle but inhibited the expression of tendon-related markers (Chen et al., 2018). Recent studies showed that senescent cells exhibited a robust increase in mRNA expression and secreted numerous bioactive factors, including degradative enzymes, inflammatory cytokines, and growth factors, which might drive stem cell dysfunction in aged stem cells through paracrine signaling (Coppe et al., 2010; Campisi et al., 2011). Whether this mechanism also accounts for age-related degenerative disorders of tendons and the factors secreted by senescent cells during tendon aging needs further research. The damage of DNA of stem cells and niche cells were hypothesized to be major contributors to inflamm-aging and the effect was propagated and amplified by macrophages (Bonafe et al., 2012). More researches are needed to verify this hypothesis in aged tendon stem cells.

## Systemic Changes of Hormonal and Metabolic Signals

Given the remarkable ability of stem cells to sense and respond to the external stimuli, it is not surprising that the age-related systemic changes t can also impact stem cells and their niches. Chronic conditions such as hormone deficiency, diabetes mellitus (DM) and hypertension are common in elderly. The systemic changes of hormonal and metabolic signals as well as drug used in the treatment of these chronic conditions can alter niche through circulation, causing stem cells to lose or deviate from their original functions.

DM has been reported to increase the prevalence/incidence of tendinopathy (Ranger et al., 2016; Lui, 2017). A recent meta-analysis of clinical studies provide strong evidence that diabetes is associated with higher risk of tendinopathy (Ranger et al., 2016), despite that there were inconsistent findings regarding the effects of DM on structural and biomechanical properties of tendon in animal models (Lui, 2017). Potential mechanisms of DM in causing and exacerbating tendinopathy by modifying stem cell functions *via* AGE formation, hyperglycemia, insulin deficiency/resistance, and adipokine dysregulation have been discussed in another review and will not be repeated (Lui, 2017).

In women, estrogen can enhance tendon collagen synthesis. Estrogen deficiency was reported to negatively affect tendon metabolism and healing. Tenocytes isolated from ovariectomised rats were reported to show lower proliferation rate, collagen type I, aggrecan, elastin, fibronectin content in the supernatant, and migration rate but higher apoptosis, VEGF and MMP-13 compared to young tenocytes (Aydin et al., 2013; Torricelli et al., 2013). Collagen content was lower in Achilles tendons after ovariectomy compared to control rats (Ramos et al., 2012). The Achilles tendons from ovariectomised rabbits also showed lower gene expressions of proteoglycans aggrecan, biglycan, decorin, and versican compared to tendons from intact normal rabbits (Huisman et al., 2014). However, there was no changes in the biomechanical properties of patellar tendon of monkeys after ovariectomy (Wentorf et al., 2006). Age-associated estrogen deficiency therefore can alter matrix composition of tendons and may influence the functions of tendon stem cells. Whether TDSCs are sensitive to estrogen is a first step to understand the influence of estrogen deficiency in age-associated loss or dysfunction of tendon stem cells.

## Potential Future Research Directions

### Research on Treatments to Reverse the Intrinsic Cellular Deficits and Aged Niche of Tendon Stem Cells

As a new cell type identified in tendon, more studies are needed to fill the gaps of the aging mechanisms of tendon stem cells. Interventions that can combat protein and DNA damages, cellular senescence, oxidative stress, chronic inflammation; and inhibit AGE formation or promote its removal may rejuvenate aged tendon stem cells. These are attractive anti-aging options to explore. The growth factor and cytokine profile of aged tendons and their effects on functions of tendon stem cells should be examined. Notably, medium of young TDSCs under hypoxic culture condition was reported to enhance the migration, proliferation and mRNA expression of tendon-related markers as well as reduced senescence of aged TDSCs (Jiang et al., 2014). Exposure of aged rat patellar tendon TDSCs to young decellularized tendon ECM enhanced their proliferation, tendon-related marker expression (*Scx*, *Tnmd*), and stem cell marker expression (Oct-4, SSEA-1) as well as reduced SA-β-gal activity (Jiang et al., 2018).

### Research on the Effects and Mechanisms of Common Aging Conditions on Tendon Stem Cells and Tendon

The effects of common aging conditions particularly diabetes, hypertension and hormonal deficiency and the drugs used on the functions of tendon stem cells and tendon should be examined. The sensitivity of TDSCs to estrogen has not been explored and should be examined. Similarly, studies on the effects of hyperglycemia, AGE, insulin deficiency/resistance, and adipokine dysregulation in diabetes on functions of tendon stem cells are needed.

### Research on the Effects and Mechanisms of Exercise on Tendon Stem Cells and Tendon

TDSCs are sensitive to mechanical load. High uniaxial cyclic tensile loading (8%) induced osteogenic differentiation of TDSCs while low uniaxial cyclic tensile loading (4%) promoted tenogenic differentiation of TDSCs (Zhang and Wang, 2010b; Rui et al., 2011; Wang et al., 2018). Modulating the mechanical load experienced by tendon stem cells therefore may counteract the effect of aging on tendon. In this regard, moderate mechanical stretching (4%) of aged TDSCs *in vitro* significantly increased the protein expression of nucleostemin and mRNA expression of *Nanog*. It also increased the expression of tenocyte-related genes (*Col1* and *Tnmd*) without increasing the expression of non-tenocyte-related genes (*Lpl*, *Sox9* and *Runx2*) of aged TDSCs (Zhang and Wang, 2015).

In an *in vivo* study, moderate treadmill running of aged mice increased the proliferation rate of aged TDSCs in culture, decreased lipid deposition, proteoglycan accumulation and calcification, and increased the expression of nucleostemin-expressing TDSCs in the patellar tendons (Zhang and Wang, 2015). Moderate exercises can also mitigate the deleterious effects of aging on the niche of tendon stem cells by improving tendon microcirculation, matrix composition, and organization as well as reducing AGE level and matrix stiffness (Couppe et al., 2014; Wood and Brooks, 2016). With training, Col1a1 and Mmp8 expression in aged mouse Achilles tendons were restored to levels similar to adult controls (Wood and Brooks, 2016). Moderate treadmill running prior to an injury augmented tendon wound healing in an aged rat model with reduced vascularity, lipid-like structures, and senescent cells as well as increased production of more collagen fibrils and better fibril organization (Zhang et al., 2016). The effect was likely mediated by the improvement of metabolism and functional fitness of aged tendon stem cells because aged TDSCs isolated from exercised rats showed higher stem cell marker expression, proliferation, colony-forming ability, and tenocyte-related gene expression as well as lower non-tenocyte-related gene expression compared to aged cells isolated from cage control rats (Zhang et al., 2016). The beneficial effect of exercise on aged tendons could also be due to systemic effects in addition to direct loading as tail tendons from aged and exercised rats assumed the elastic characteristics of their younger counterparts after exercise (Lacroix et al., 2013). Stem cells expressed different channels that could sense membrane tension (Soria et al., 2013). One study showed that the stretch-mediated activation channel, cystic fibrosis transmembrane conductance regulator (CFTR), was up-regulated in TDSCs during tenogenic differentiation under mechanical stretching (Liu et al., 2017). Further studies to confirm the beneficial effects, optimal loading protocol, and mechanosensing mechanisms of excise on aged tendon stem cells and tendon should be done.

## Conclusion and Future Perspectives

**Figure 1:**
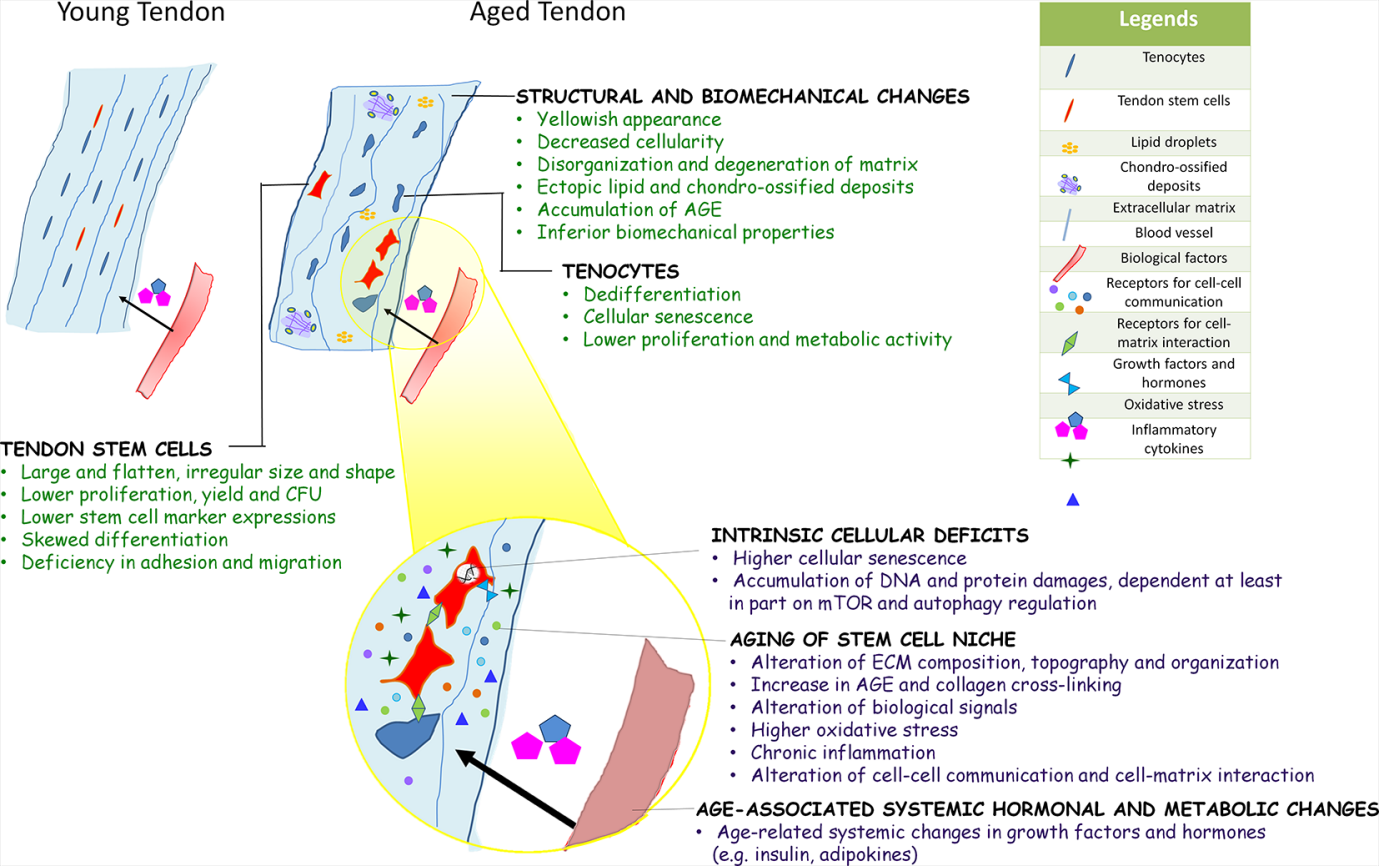
Diagram summarizing the key biological characteristics of aged tendon, and potential factors influencing the fate and functions of TDSCs during tendon aging. Current evidences on aging of other adult stem cells and limited data on tendon-derived stem cells (TDSCs) suggested that aging of tendon stem cell likely occurs *via* intrinsic cellular deficits, aging of stem cell niche, and age-associated systemic hormonal and metabolic changes. These factors interact with each other to affect stem cell function at multiple levels.

In summary, the composition, structure, and mechanical properties of tendons deteriorate with aging and are driven at least in part by an age-associated decline in the metabolism and functional fitness of tendon stem cells. Unlike their young and healthy counterparts, aged TDSCs were shown to be larger and flatten. The number and stemness of TDSCs isolated from aged tendons were lower. They exhibited altered multi-lineage differentiation potential, deficiency in cell adhesion, and migration as well as early cellular senescence. The intrinsic cellular deficits, aged niche, and systemic hormonal and metabolic changes associated with aging can drive the aging process of stem cells, some of which have been demonstrated in TDSCs recently. There is no evidence about the factor(s) triggering the aging of tendon stem cells. The authors speculate that they may work together in inducing functional deficits of tendon stem cells as aging is a continued process. **Figure 1** summarizes the key biological characteristics of aged tendon, and potential factors influencing the fate and functions of TDSCs during tendon aging. Uncovering the mechanisms underpinning the regenerative decline of metabolism and functions of aged tendon stem cells would have profound implications for the development of stem cell-based therapeutics to reset the aging clock of tendons.

## Author Contributions

PL conceived the idea, wrote, and proof-read the manuscript. CW wrote and proof-read the manuscript.

## Funding

This study was supported by the one-line budget of The Hong Kong Polytechnic University (SF1920-CM).

## Conflict of Interest

The authors declare that the research was conducted in the absence of any commercial or financial relationships that could be construed as a potential conflict of interest.
